# Supplementary figures and images for: Preclinical Evaluation of the FGFR-Family Inhibitor Futibatinib for Pediatric Rhabdomyosarcoma
Source: Cancers (Basel). 2023 Aug 9;15(16):4034. doi: 10.3390/cancers15164034 (PMC10452847; doi:10.3390/cancers15164034)

**Figure 1A**

p-FGFR4

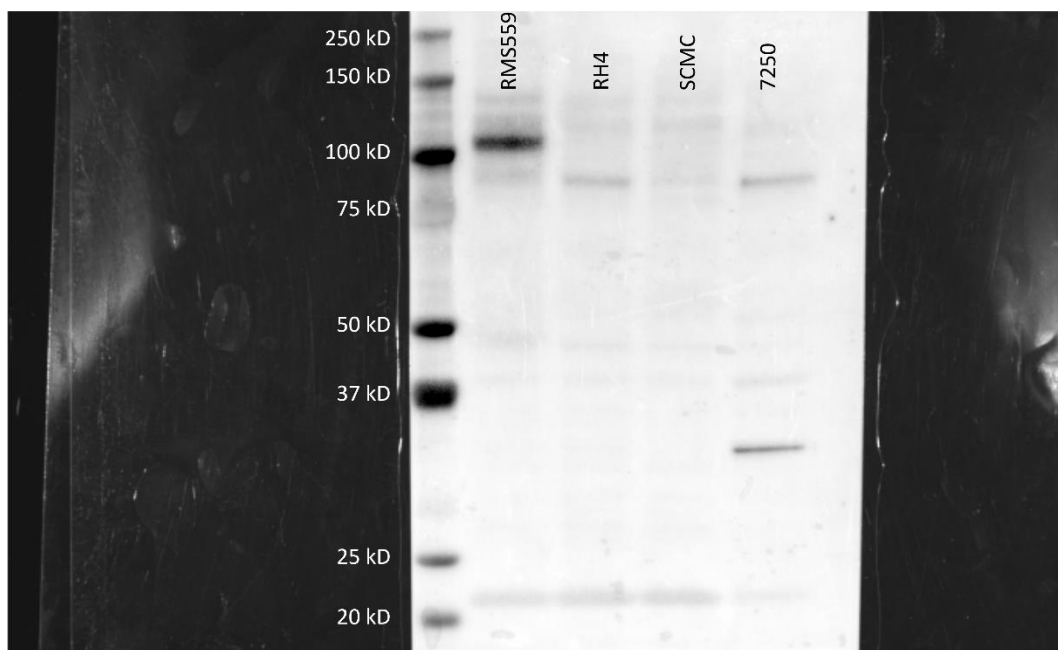

FGFR4

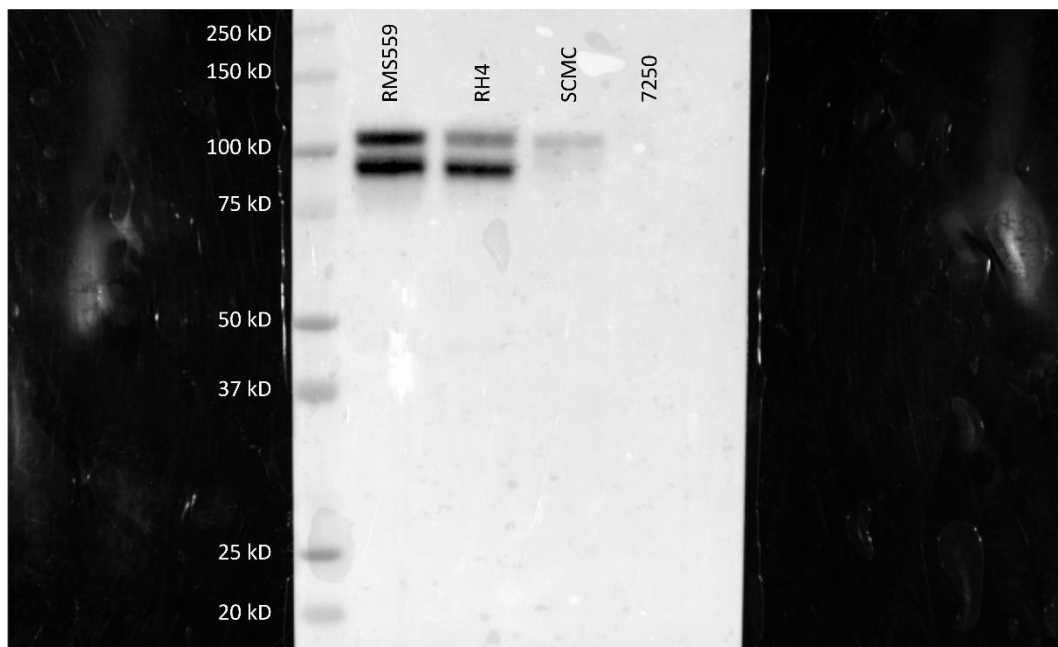

$\beta$ -Actin

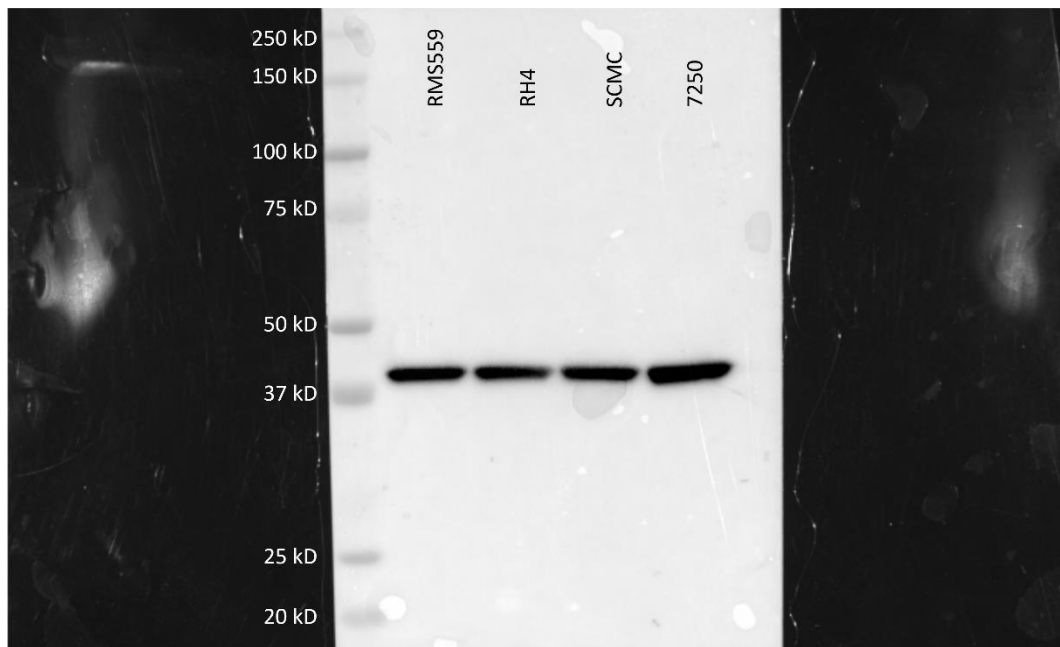

**Figure 1D**

p-FGFR4

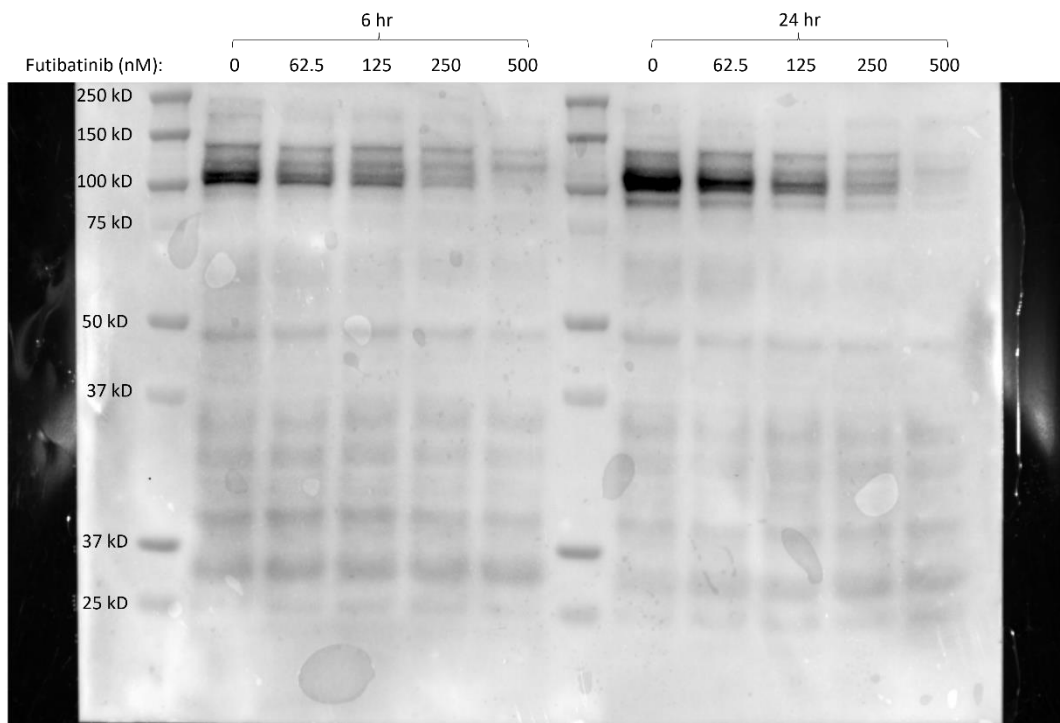

FGFR4

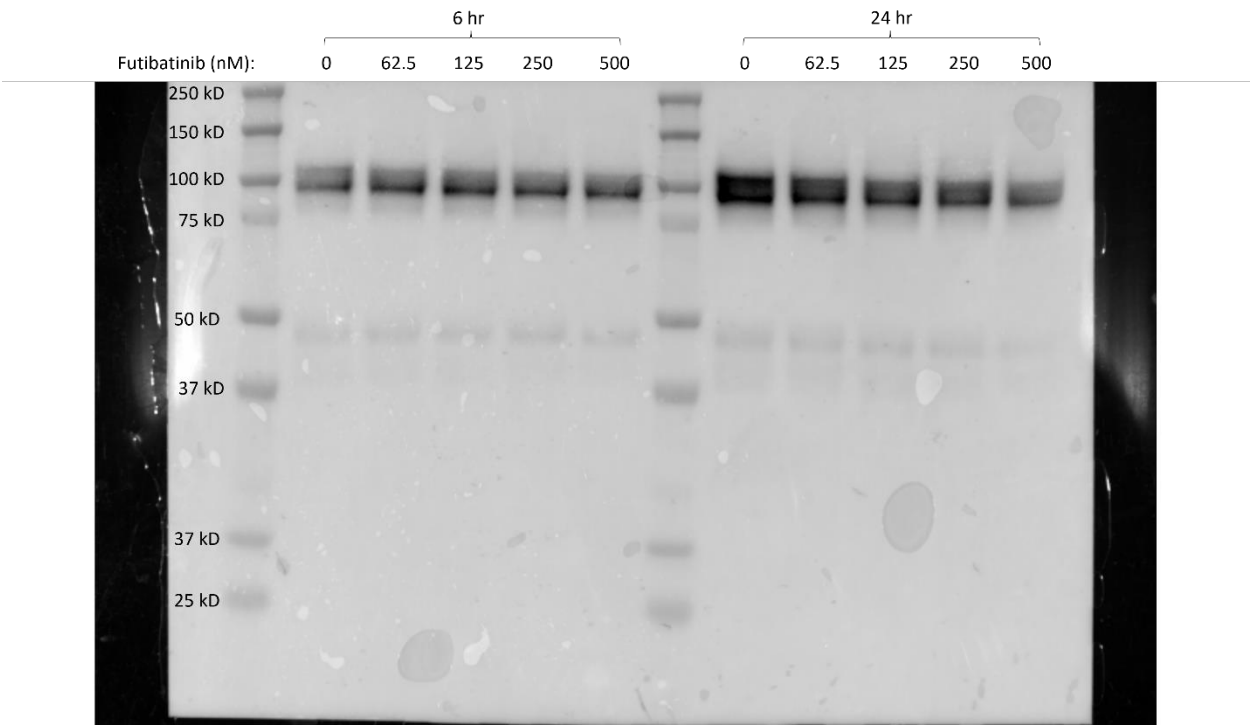

p-ERK1/2

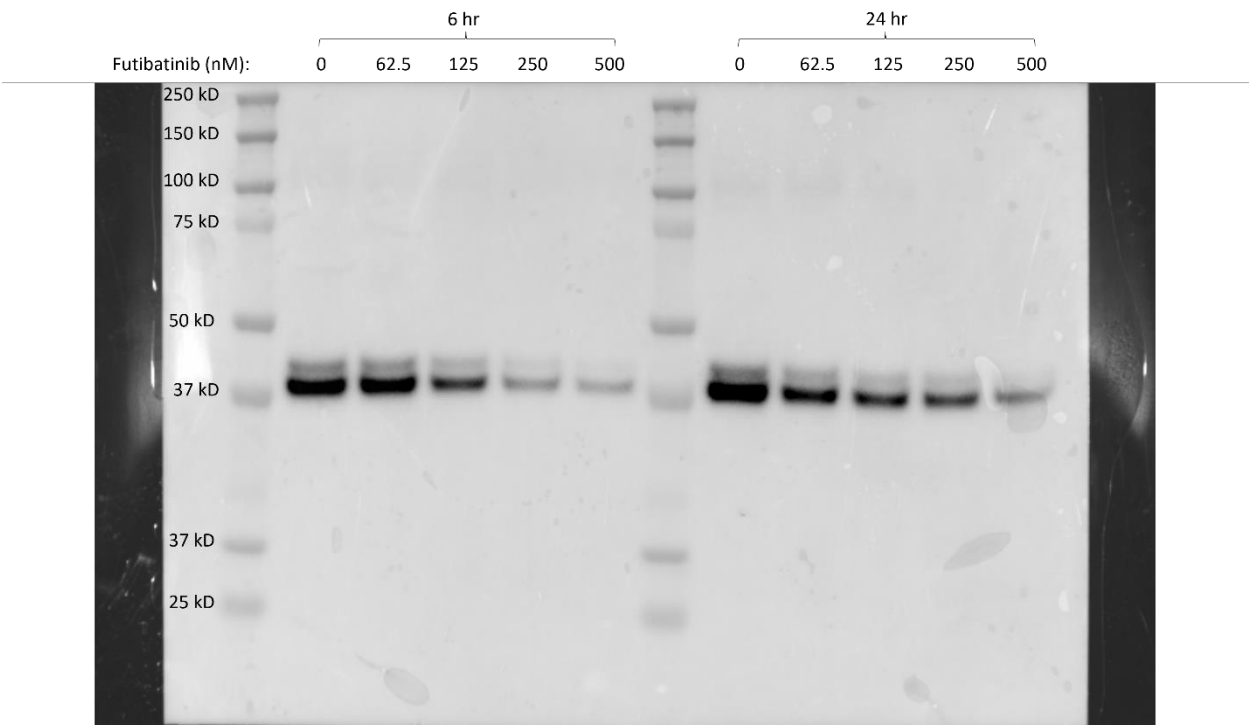

ERK1/2

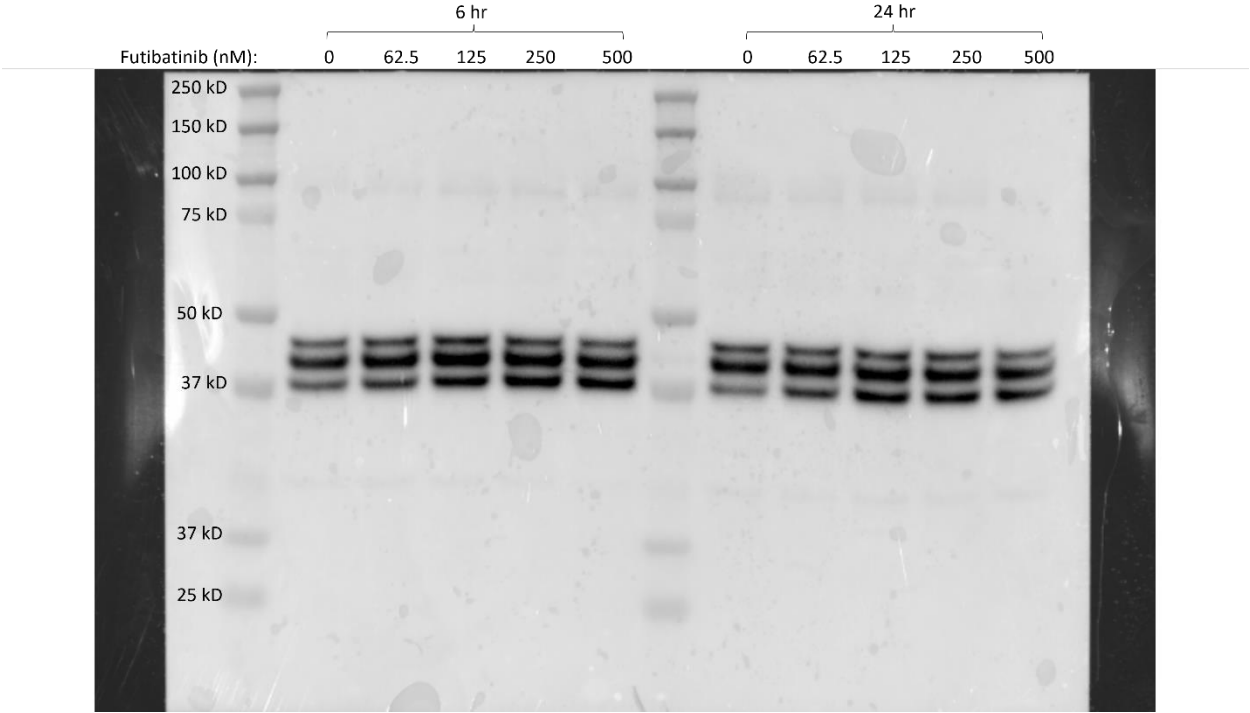

GAPDH

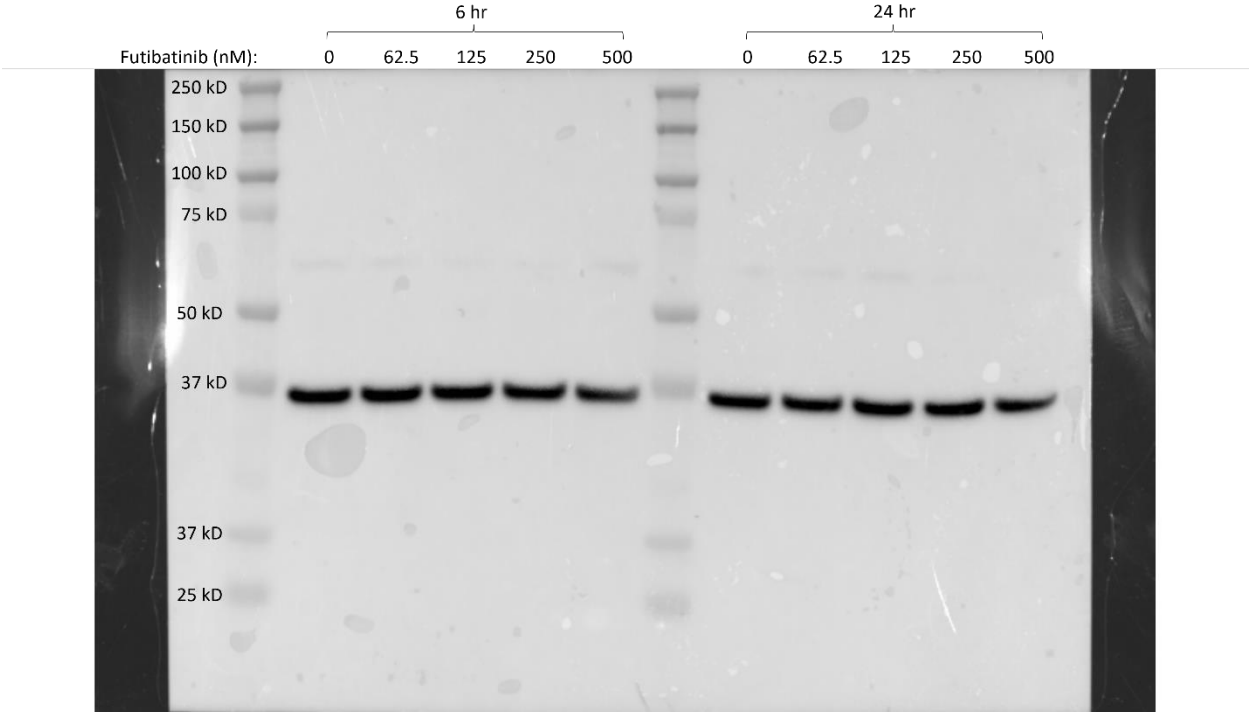

Supplement: Supplementary file 1 [file cancers-15-04034-s001.zip › cancers-2442190-supplementary.pdf]
